# *Bacillus megaterium* Has Both a Functional BluB Protein Required for DMB Synthesis and a Related Flavoprotein That Forms a Stable Radical Species

**DOI:** 10.1371/journal.pone.0055708

**Published:** 2013-02-14

**Authors:** Hannah F. Collins, Rebekka Biedendieck, Helen K. Leech, Michael Gray, Jorge C. Escalante-Semerena, Kirsty J. McLean, Andrew W. Munro, Stephen E. J. Rigby, Martin J. Warren, Andrew D. Lawrence

**Affiliations:** 1 School of Biosciences, University of Kent, Canterbury, Kent, United Kingdom; 2 Institute of Microbiology, Technical University Braunschweig, Braunschweig, Germany; 3 Department of Bacteriology, University of Wisconsin, Madison, Wisconsin, United States of America; 4 Manchester Interdisciplinary Biocentre, University of Manchester, Manchester, United Kingdom; Louisiana State University and A & M College, United States of America

## Abstract

Despite the extensive study of the biosynthesis of the complex molecule B_12_ (cobalamin), the mechanism by which the lower ligand 5,6-dimethylbenzimidazole (DMB) is formed has remained something of a mystery. However, recent work has identified and characterized a DMB-synthase (BluB) responsible for the oxygen-dependent, single enzyme conversion of FMN to DMB. In this work, we have identified BluB homologs from the aerobic purple, nonsulfur, photosynthetic bacterium *Rhodobacter capsulatus* and the aerobic soil bacterium *Bacillus megaterium* and have demonstrated DMB synthesis by the use of a novel complementation assay in which a B_12_ deficient strain, substituted with the precursor cobinamide is recovered either by the addition of DMB or by the recombinant expression of a *bluB* gene. The DMB-synthetic activity of the purified recombinant BluB enzymes was further confirmed *in vitro* by providing the enzyme with FMNH_2_ and oxygen and observing the formation of DMB by HPLC. The formation of a 4a-peroxyflavin intermediate, the first step in the oxygen dependent mechanism of DMB biosynthesis, is reported here and is the first intermediate in the enzyme catalysed reaction to be demonstrated experimentally to date. The identification and characterization of an FMN-binding protein found on the *cobI* operon of *B. megaterium*, CbiY, is also detailed, revealing an FMN-containing enzyme which is able to stabilize a blue flavin semiquinone upon reduction with a 1-electron donor.

## Introduction

5,6-Dimethylbenzimidazole (DMB), a derivative of benzimidazole, forms the alpha-axial ligand of cobalamin and is the critical distinguishing factor between cobalamin and pseudocobalamin (in which an N7-linked adenine forms the lower ligand) [1], [2]. There has historically been an interest in differentiating between the biosynthesis of cobalamin and pseudocobalamin since, as the name suggests, the latter cannot be used by humans, whilst the former is an important dietary cofactor [3].

Earlier research by Renz [4] had shown that at least two pathways for DMB synthesis exist. The first of these is an aerobic pathway that sees the transformation of FMN into the base. Here it has long been known that flavin mononucleotide (FMN) is a substrate in the *de novo* biosynthesis of DMB in B_12_-producing micro-organisms [5] and that the C2 carbon of DMB is derived from the C1′ carbon of the flavin [6]. It has only relatively recently been shown that a single protein, BluB, is responsible for the complete conversion of reduced FMN into DMB [1], [2]. An alternative anaerobic route also exists where several building blocks including erythrose, formate, glutamine, glycine, and methionine are required for the synthesis of the base [4].

In the aerobic biosynthesis the formation of DMB from flavin is a complex process involving the contraction of the isoalloxazine ring system and cleavage of the ribityl tail [6], [7]. This transformation, for which no enzymological precedent exists, sees the destruction of the flavin and production of DMB along with the predicted by-products urea and erythrose-4-phosphate [6], [8] and gives rise to the suggestion that BluB could be categorized as a ‘flavin destructase’.

Intriguingly, the enzyme displays a quite unusual requirement for reduced FMN as a substrate but also requires oxygen for the formation of DMB to proceed [1]. The BluB from *Sinorhizobium meliloti* was the first to be characterized and the crystal structure solved [1]. The crystal structure revealed that the active site of BluB is relatively small and sandwiched between the two units of a dimer. This diminutive active site is thought to contribute to the selectivity of the enzyme for molecular oxygen [1], [2]. Despite the requirement for a reduced flavin substrate, the enzyme is not itself a flavin reductase, but rather it seems likely that the flavin is reduced independently elsewhere, before delivery to BluB [1].

Cobalamin biosynthesis is mediated by one of two pathways, either by a late cobalt insertion (aerobic) pathway or by an early cobalt insertion (anaerobic) pathway. The differences between the pathways are in the timing of the insertion of the cobalt and mechanism of ring contraction [9]. The BluB proteins studied to date have been derived from organisms utilising the late cobalt insertion pathway [1], [2], [10]. In this study we have chosen to isolate DMB-synthases from bacterial species utilising each of these pathways. Thus, the organisms we have selected, *B. megaterium* and *R. capsulatus*, represent the early and late cobalt insertion pathways respectively [11], [12]. By identifying the BluB orthologs from these established examples of the two pathways of B_12_ biosynthesis we have been able to demonstrate that BluB is not specific to the late cobalt insertion pathway. Rather, we have been able to isolate BluB from each pathway and shown that both are single-enzyme DMB-synthases, and that both require oxygen to catalyze the conversion of FMN into DMB.

## Results

### Identification of a BluB Homologue in *Bacillus Megaterium*

The amino acid sequences of three BluB proteins from organisms harbouring the oxygen-dependent pathway towards cobalamin, *Rhodobacter capsulatus* [13], *Rhodospirillum rubrum* [2] and *Sinorhizobium meliloti* [1] were used as search templates to mine the genome of *B. megaterium* (strain DSM319). Two potential gene candidates, (BMD_2595 and BMD_0969) were identified. The first, BMD_2595, also known as *cbiY*, is located at the end of the *cobI* operon [11] and displays a sequence identity of between 18 to 20% to the other BluB enzymes. BMD_0969, which shall be refered to as BluB_(Bm)_ hereafter, is not located in proximity to any genes known to be involved in cobalamin biosynthesis and displays a higher sequence identity of between 29 to 40% to the other *bluB* templates (Table S1). In *R. capsulatus* the *bluB* gene is located in a large operon that codes for the majority of genes responsible for the biosynthesis of cobalamin [14]. To investigate the activity of these two proteins, the *bluB_(Bm)_* and *cbiY_(Bm)_* genes together with the *bluB_(Rc)_* gene of *R. capsulatus* were individually amplified by PCR and cloned into pET14b for recombinant overproduction of the encoded products as N-terminal His-tag fusions in *E. coli*.

### Complementation of BluB Activity in *Salmonella Enterica*

*S. enterica* is able to facilitate the corrinoid-dependent utilization of ethanolamine in the presence of vitamin B_12_ under aerobic conditions, thus enabling its growth on ethanolamine enriched media if vitamin B_12_ is available. However it can make vitamin B_12_ from exogenous cobinamide only if DMB is also provided [15], [16].

**Figure 1:**
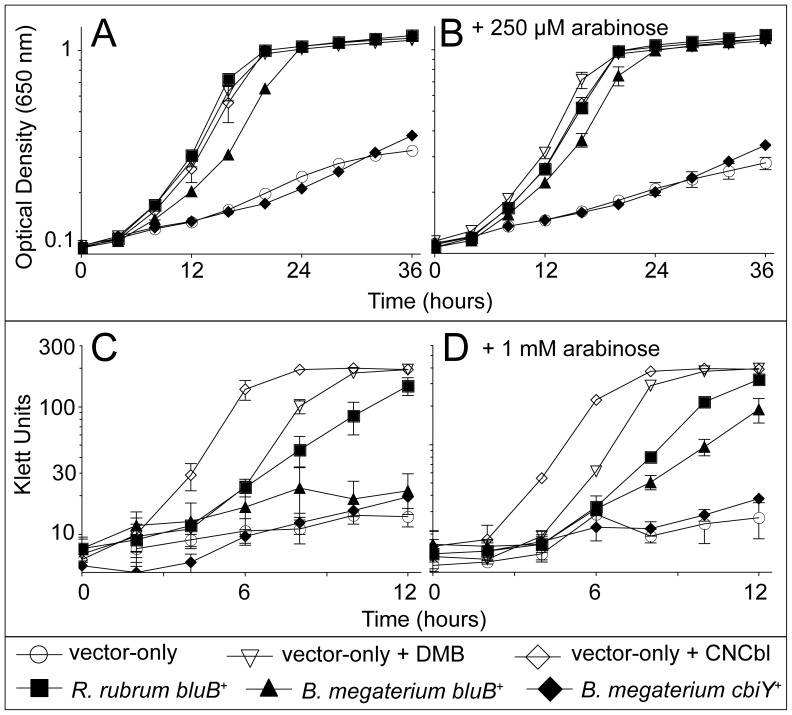
The *bluB* gene of *Bacillus megaterium* encodes DMB synthase. The aerobic growth of *Salmonella enterica* strain JE7087, carrying pBAD24 (vector-only, open circles), pBluB9_(Rr)_ (*R. rubrum BluB* ^+^; black squares), pBluB25_(Bm)_ (*B. megaterium bluB* ^+^; black triangles), and pCbiY6_(Bm)_ (*B. megaterium cbiY* ^+^; black diamonds) in NCE minimal medium containing 90 mM ethanolamine, 1 mM MgSO_4_, 1× trace minerals [41], 50 µg µl^−1^ ampicillin, and 150 nM dicyanocobinamide. Where indicated, cobinamide was replaced with vitamin B_12_ (open diamonds), DMB was added at 30 µM (open triangles), and arabinose was added at 250 µM (Panel B). Optical density at 650 nm was measured for 36 h at 37°C. Growth curves were obtained using an ELx808 Ultra Microplate reader (Bio-Tek Instruments). Each growth curve was performed in triplicate, and error bars of one standard deviation are indicated. **Panels C and D** show aerobic growth of *S. enterica* strain JE2119 (*S. enterica metE205 ara-9 cobC1175*::Tn10d[*tet* ^+^]) carrying pBAD24 (vector-only, open circles), pBluB9_(Rr)_ (*R. rubrum bluB* ^+^; black squares), pBluB25_(Bm)_ (*B. megaterium bluB* ^+^; black triangles), and pCbiY6_(Bm)_ (*B. megaterium cbiY* ^+^; black diamonds) in NCE minimal medium containing 11 mM glucose, 1 mM MgSO_4_, 1× trace minerals (Balch and Wolfe 1976), 50 µg µl^−1^ ampicillin, and 15 nM dicyanocobinamide. Where indicated, dicyanocobinamide was replaced with cyanocobalamin (open diamonds), DMB was added at 30 µM (open triangles), and arabinose was added at 500 µM (Panel D). Aerobic growth of JE2119 derivatives took place in 5 ml volumes in 125 ml sidearm flasks which were shaken at 280 rpm at 37°C. Growth was monitored with a Summerson colorimeter (Klett). Each growth curve was performed in triplicate, and error bars of one standard deviation are indicated.

Cobinamide is a coenzyme B_12_ precursor that lacks the lower nucleotide loop (Nomenclature, I.–I. C. o. B., 1974). *S. enterica* strain JE9385 carrying the empty cloning vector pBAD24 is unable to grow on ethanolamine in the presence of 150 nM cobinamide (Figure 1A & B, open circles) in the absence of exogenous DMB. Addition of 30 µM DMB (Figure 1A & B, open triangles) or replacement of the cobinamide with 150 nM vitamin B_12_ (Figure 1A & B, open diamonds) allows growth under these conditions. This strain was used in a complementation bioassay to detect the production of DMB.

In order to determine which of the genes from *B. megaterium* is a DMB synthase, *R. rubrum bluB* [2], *cbiY* from *B. megaterium*, and *bluB* from *B. megaterium* were cloned into pBAD24 [17] under the control of the arabinose-inducible P_BAD_ promoter to yield pBluB9_(Rr)_, pCbiY6_(Bm)_, and pBluB25_(Bm)_, respectively. When transformed into JE9385 the *R. rubrum bluB* gene, known to encode DMB synthase [2], allowed strong growth on ethanolamine both when uninduced and when induced by the addition of arabinose (Figure 1A & B, closed squares). Transformation of JE9385 with the *B. megaterium bluB* gene construct permitted growth (Figure 1B, closed triangles), although in the absence of arabinose this growth was slightly delayed (Figure 1A, closed triangles). The *B. megaterium cbiY* gene did not allow growth of JE9385 on ethanolamine at any tested arabinose concentration (Figure 1A & B, black diamonds).

Further confirmation that the *B. megatarium* BluB encodes a DMB synthase was achieved in a second bioassay. Here a *S. enterica metE cobC* double mutant was employed as a reporter for cobalamin as under highly aerobic conditions, this mutant strain cannot grow in glucose minimal medium with 15 nM cobinamide unless provided with exogenous DMB [18] (Figure 1C & D, open circles and open triangles). In contrast replacement of cobinamide with vitamin B_12_ resulted in rapid growth (Figure 1C & D, open diamonds). Transformation of this strain with the *R. rubrum BluB* gene permitted relatively slow growth (Figure 1C, black squares), while the *B. megaterium BluB* gene was unable to rescue any growth (Figure 1C, black triangles). Addition of 1 mM arabinose allowed growth of the strain containing the *B. megaterium bluB* (Figure 1D, black triangles), but had no significant effect on the growth of the strain containing *bluB* from *R. rubrum* (Figure 1D, black squares). The *B. megaterium cbiY* gene did not allow growth of a *metE cobC* mutant at any tested arabinose concentration (Figure 1C & D, black diamonds).

### Purification of Recombinant BluB and CbiY Enzymes

**Figure 2:**
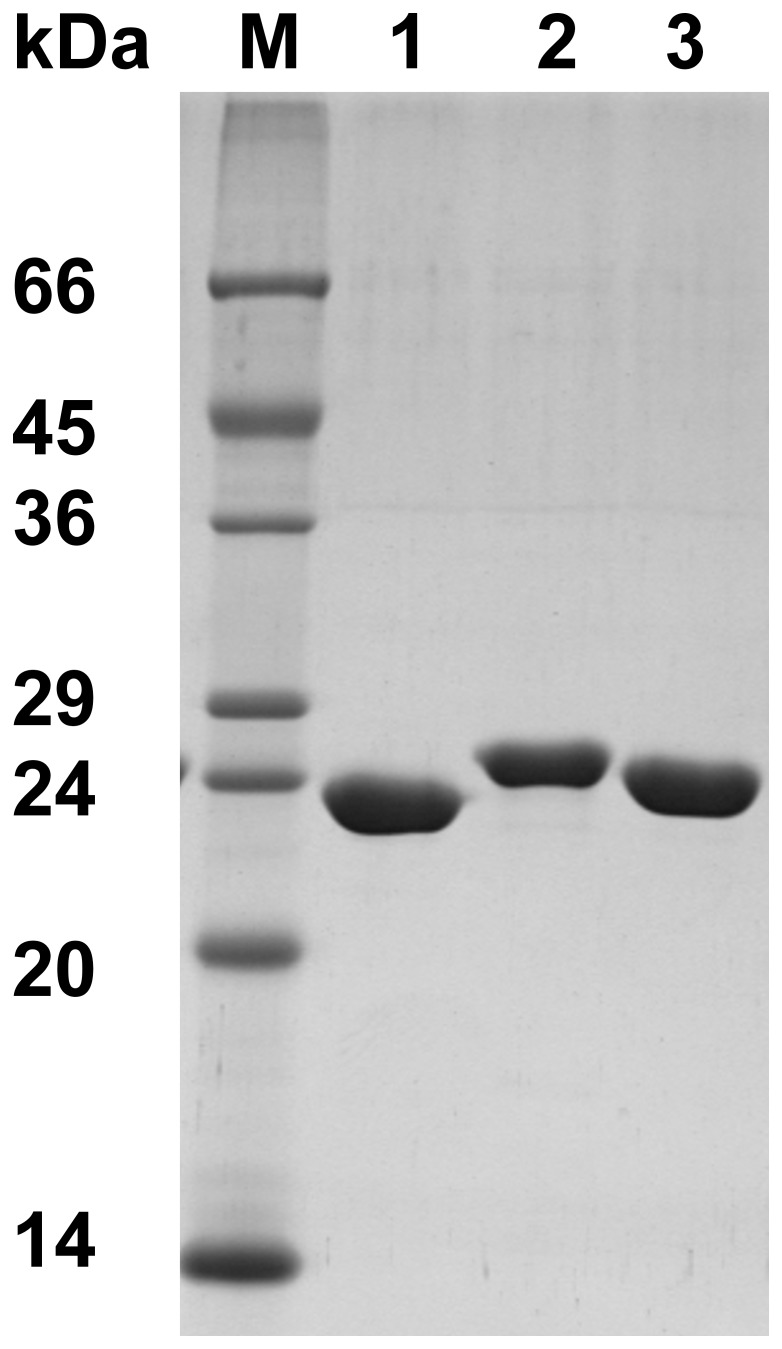
SDS-PAGE gel of purified BluB and CbiY enzymes. 12.5% SDS gel showing the recombinantly overproduced and purified His_6_-tagged fusion proteins BluB_(Rc)_ (lane 1), BluB_(Bm)_ (lane 2), and CbiY_(Bm)_ (lane 3) with their relative molecular weights of 25,000, 27,300, and 25,800, respectively.

The *B. megaterium* BluB and CbiY proteins and the *R. capsulatus* BluB were overproduced in *E. coli* with N-terminal His-tags and purified by metal affinity chromatography under aerobic conditions. SDS-PAGE analysis showed all three fusion proteins migrated as single bands with molecular masses between 25 to 27.3 kDa (25 kDa for BluB_(Rc)_, 27.3 kDa for BluB_(Bm)_, and 25.7 for CbiY_(Bm)_) (Figure 2). While the purified fractions of both BluB proteins (at concentrations of up to 904 µM) were colourless, fractions containing CbiY_(Bm)_ (100 µM) were found to be bright yellow.

### *In vitro* Dimethylbenzimidazole (DMB) Synthase Activity

**Figure 3:**
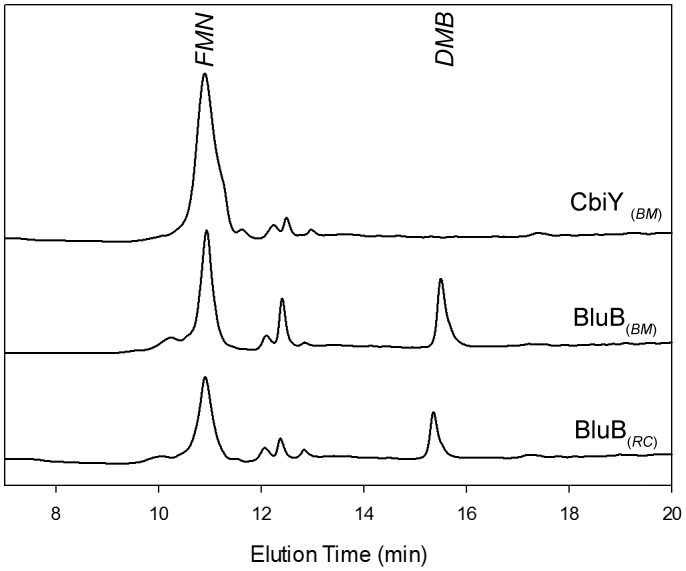
HPLC analysis of reaction products from *in vitro* activity assays using BluB_(Rc)_, **BluB_(Bm)_ and CbiY_(Bm)_.** The reactions were prepared in buffer A at ambient temperature with 100 µM FMN, 8 mM NADH, and 1 mM DTT, and contained either 1.47 mM BluB_(Rc)_, 1.50 mM BluB_(Bm)_, or 1.38 mM CbiY_(Bm)_, respectively. Before HPLC analysis, the reaction mixture was heated to 100°C to denature the enzymes.

DMB synthase activity was also investigated using an *in vitro* HPLC based assay. The purified proteins were buffer exchanged (PD-10 column) and incubated with 100 µM FMN and 8 mM NADH. Protein concentrations ranged from 1 to 500 µM. NADH is required to reduce FMN to FMNH_2_ which is the substrate for the BluB reaction. The reaction was incubated in the presence of oxygen [2], [19] for 18 hours. Following this, the reaction mixture containing CbiY_(Bm)_ remained bright yellow and was comparable to the control that contained no enzyme. However, incubations containing BluB_(Bm)_ and BluB_(Rc)_ were pale yellow in colour. The products of the reaction were analyzed by HPLC by comparison to authentic standards of DMB and FMN. DMB formation was observed in reaction mixtures containing either BluB_(Rc)_ or BluB_(Bm),_ whereas no DMB could be detected in incubations containing CbiY_(Bm)_ (Figure 3). Quantification of the amount of DMB formed during the reaction revealed that the *R. capsulatus* enzyme was slightly more active than the *B. megaterium* BluB. No DMB could be detected if the reaction mix was incubated in the absence of oxygen. These findings indicate that the BluB enzyme from *B. megaterium*, which is known to use the early cobalt insertion (anaerobic) pathway for adenosylcobalamin synthesis, has a requirement for oxygen to complete the biosynthesis of cobalamin.

### BluB Forms a Peroxyflavin Intermediate

**Figure 4:**
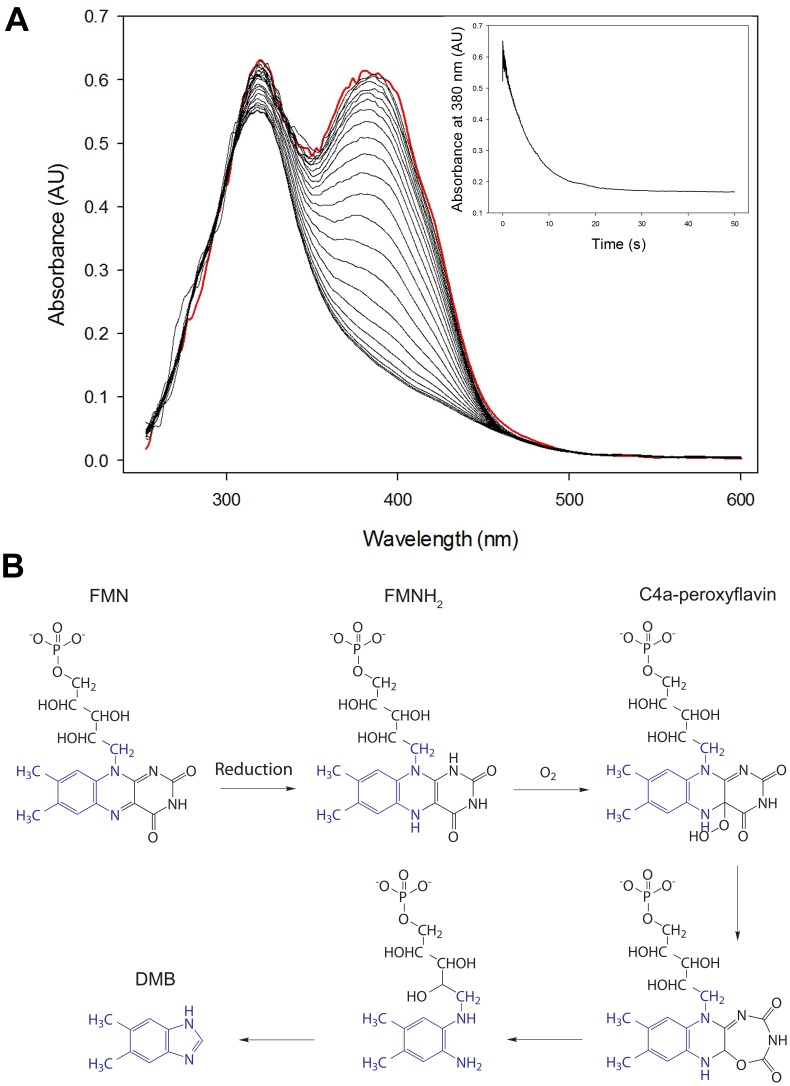
Formation of BluB peroxyflavin intermediate. (**A**) 291 µM FMNH_2_ was incubated with 684 µM (RC)BluB and prior to stopped flow mixing with oxygenated buffer A. The formation of a c4a-peroxyflavin absorbance peak at 380 nm is complete in less than 2.5 s (red) and is followed by a gradual loss of absorbance demonstrated by the black spectra recorded every 2.5 s. The inset shows the formation and decay of the peroxyflavin, represented by absorbance at 380 nm, as a function of time. (**B**) Reaction scheme of the DMB formation mechanism. The first reaction step is catalyzed by a FMN reductase (Taga *et al.* 2007) which provides the substrate FMNH_2_ for BluB, while the next four steps are catalysed by BluB.

BluB catalyzes the O_2_-dependent conversion of FMNH_2_ to DMB. The first step in the proposed mechanism (Figure 4) is the activation of molecular oxygen forming the peroxyflavin intermediate. To confirm the presence of such an intermediate in the BluB catalysed reaction, FMNH_2_ (formed from the chemical reduction of FMN with sodium dithionite), which is the substrate for BluB [1], [2], was incubated with different ratios of purified BluB from *R. capsulatus* or *B. megaterium* in an anaerobic chamber. Peroxyflavin formation was initiated by rapidly mixing the protein solution containing FMNH_2_ with buffer containing oxygen in a stopped-flow apparatus and the reaction was monitored using a multi-wavelength diode array detector. For this purpose, oxygenated buffer (equilibrated in atmospheric air) was sealed in a syringe and taken into the anaerobic chamber. In the case of the BluB_(Rc)_ enzyme, rapid formation of a peroxyflavin intermediate, with an absorption maximum at 380 nm, was observed (Figure 4). The absorbance peak at 320 nm can be attributed to sodium dithionite (used to reduce the flavin prior to beginning the stopped flow experiment). This peak is also present in the control spectra (Figure S1), and as such cannot be attributed to the formation of a stable intermediate. It is possible to calculate the theoretical spectrum of the peroxyflavin by subtracting the control spectrum from the spectrum observed experimentally (Figure S2). The initial peroxyflavin formation step was complete within 20 ms of mixing and was followed by a much slower decay of the optical signal (in the order of seconds) which seems to occur in a biphasic manner, this is possibly due to there being both productive and non-productive decay of the peroxyflavin (Figure 4). The apparent rate of peroxyflavin formation and decay was unchanged over a range of enzyme concentrations (Figure S3). Diminishing oxygen concentration, accomplished by re-equilibration of the oxygen containing buffer in the anaerobic chamber, had little effect on the rate of formation. However, under these conditions no peroxyflavin could be detected with *B. megatarium* BluB.

### Characterisation of CbiY_(Bm)_

Following the results of the *in vitro* and microbiological DMB synthase assays, the function of CbiY remained to be determined. In order to investigate the role it may play within the biosynthesis of cobalamin it was decided to characterize the protein further. Unlike the BluB enzymes from *R. capsulatus* and *B. megaterium*, fractions containing purified CbiY were bright yellow in colour, indicating that the protein binds a flavin cofactor. The UV-visible spectrum of the protein confirmed the presence of a flavin with absorbance maxima at 362 and 450 nm and shoulders at 420 and 472 nm. To distinguish between FMN and FAD, the cofactor was removed from the protein by heat denaturation and subjected to analysis by HPLC. The retention time of the extracted flavin was consistent with the bound cofactor being FMN (data not shown).

Flavoproteins are typically involved in electron transfer processes, and often interact with NAD(P)(H) coenzymes. On addition of either NADH or NADPH to CbiY under anaerobic conditions, the yellow colour of the CbiY FMN cofactor was bleached, consistent with the 2e^−^ reduction of the flavin to its hydroquinone state. When the protein was titrated with a one electron reducing agent (sodium dithionite) the reduction of the flavin proceeded through a semiquinone intermediate, as observed by the development of a broad absorption band centred at ∼600 nm which is characteristic of a neutral (blue) semiquinone. The midpoint reduction potentials for the FMN cofactor housed within CbiY were determined by subjecting the protein to a spectroelectrochemical potentiometric titration using sodium dithionite as the reductant and potassium ferricyanide as the oxidant. The protein remained stable for the duration of the titration and no hysteresis was observed between titrations in oxidative and reductive directions.

**Figure 5:**
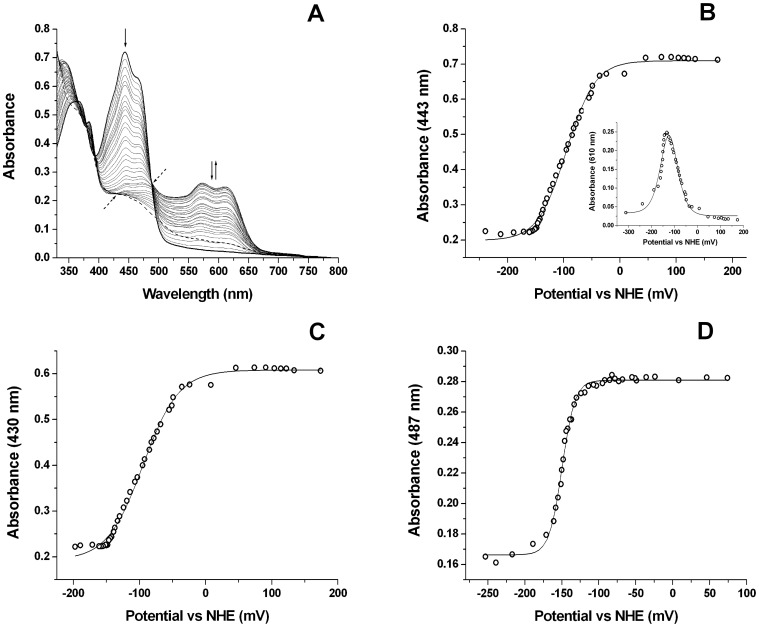
Determination of the CbiY flavin midpoint redox potentials. CbiY in 100 mM sodium phosphate buffer pH 7.0 containing 10% glycerol was subjected to a redox titration using dithionite as the reductant and ferricyanide as the oxidant under anaerobic conditions. Panel (A) shows selected spectra from the redox titration of CbiY (60 µM), showing the progressive bleaching of the oxidized flavin spectrum (A_443_ decrease indicated by solid down-arrow) on reduction, accompanied by accumulation and then decay of the blue semiquinone species (∼A_610_ indicated by solid up- and down-arrows) as the first and then second electrons are added to the flavin. Dashed arrows indicate isosbestic points for the ox/sq (*E*_1_, 487 nm) and sq/hq (*E*_2_, 430 nm) transitions. Panel B shows a plot of A_443_ data (near the oxidized flavin maximum) versus applied potential, with data fitted using a 2-electron Nernst function to produce midpoint redox potential values (vs. the normal hydrogen electrode, NHE) of *E*_1_ = −91±4 mV and *E*_2_ = −142±10 mV. The inset shows fitting of data at the CbiY semiquinone absorption maximum (A_610_) to produce values of E1 = −95±4 mV and *E*_2_ = −153±8 mV. Panel B show a data fit at the sq/hq isosbestic point (430 nm), yielding a value of *E*_1_ = −92±5 mV, while panel D shows the respective data fit at the ox/sq isosbestic point (487 nm), yielding a value of *E*_2_ = −149±3 mV.

Absorption versus potential data were fitted at different wavelengths in order to obtain robust estimates for the midpoint potentials of the CbiY FMN ox/sq (*E*_1_) and sq/hq (*E*_2_) transitions, as well as for the overall 2-electron reduction of the FMN (ox/hq, *E*_12_). The CbiY FMN was essentially completely converted from fully oxidized to completely hydroquinone in the range from 0 to −200 mV versus the normal hydrogen electrode (NHE). Figure 5A shows spectra from the redox titration, demonstrating the progressive decrease in absorption at the major flavin band during reduction of the flavin, and on the increase and subsequent decrease in the absorption feature centred at ∼600 nm reporting on the formation and decay of a neutral (blue) semiquinone. Data fitting at 610 nm (near the peak of the blue semiquinone) to a 2-electron Nernst function provided data consistent with those from analysis at the isosbestic points, with *E*_1_ = −95±4 mV and *E*_2_ = −153±8 mV (Figure 5B inset). Data fitting using the same function at a wavelength near the oxidized flavin maximum (443 nm) also provided midpoint potential values for the *E*_1_ (−91±4 mV) and *E*_2_ (−142±10 mV) couples that were consistent with the other data sets (Figure 5B).

A much smaller peak at ∼385 nm shows a similar pattern of formation and decay, and possibly reflects a small proportion of anionic (red) semiquinone. Clear isosbestic points are observed at 487 nm and 430 nm for the ox/sq and sq/hq couples, respectively. Data fitting at these wavelengths provides values for *E*_2_ (sq/hq) = −149±3 mV, and for *E*_1_ (ox/sq) = −92±5 mV, respectively (Figures 5C and 5D).

**Figure 6:**
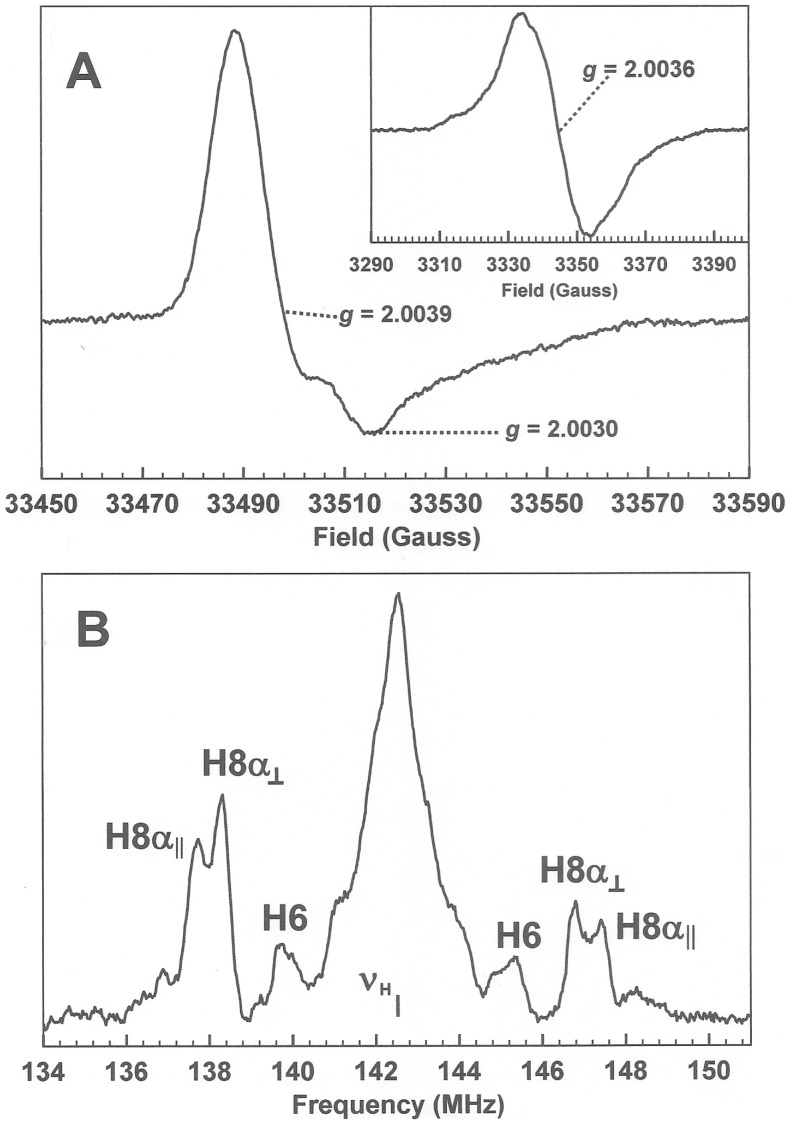
ENDOR spectrum of the CbiY bound flavin radical. (A) W-band (94 GHz) cw-EPR spectrum of the CbiY flavin radical. Experimental parameters: microwave power 65 nW, modulation frequency 100 kHz, modulation amplitude 1 G, temperature 120 K. Inset is the spectrum of the CbiY flavin radical recorded at X-band EPR (B) W-band Davies pulsed ENDOR spectrum of the CbiY bound flavin semiquinone radical recorded at t = 120 K. The pulse sequence π-T-π/2-τ-π-acq was employed with π = 400ns, T = 20 µs and τ = 1.2 µs. A 18 µs radiofrequency pulsed was applied during T, 1 µs after the initial π pulse. The field was set to g = 2.0039 (33498 G).

The formation of a neutral semiquinone radical was further confirmed by EPR and ENDOR studies of the one electron reduced protein at X- and W-band. For EPR samples, CbiY (between 1 and 10 mM in 100 mM phosphate buffer pH 7.0 containing 10% glycerol) was anaerobically reduced with sodium dithionite until the proportion of semiquinone reached its maximum. The protein was then transferred into quartz EPR tubes and frozen in liquid nitrogen. The X-band cw-EPR spectrum was recorded at 100 K and exhibited a line-width of 19 G and a g_av_ of 2.0036 which is consistent with the formation of a neutral flavin semiquinone radical [20]. The same sample at W-band gave rise to the axial EPR spectrum of Figure 6A. The apparent g values marked on the figure would give a gav of 2.0036, however, it is known from studies at yet higher fields that flavin semiquinones display rhombic g anisotropies which can only be determined by spectrum simulation [21]. As neither EPR spectrum of the protein bound flavin radicals displays resolved hyperfine structure, due to the anisotropies and multiplicities of the hyperfine interactions [20], [21], the Davies pulsed ENDOR spectrum [22] of the radical was acquired at W-band. The spectrum, Figure 6B, shows several prominent features which arise through the coupling of protons covalently attached to the 7,8-dimethyl isoalloxazine ring and are consistent with previous studies of neutral flavosemiquinone radicals [23]. The most intense feature is the matrix-ENDOR signal in the middle of the spectrum which extends from around 140.4 to 144.4 MHz and is centred on the free proton Larmor frequency. This signal arises from an overlap of multiple resonances with only small hyperfine couplings that originate from protons surrounding the flavin in the binding pocket, either protein protons or water protons along with some weakly coupled protons from the isoallozazine ring [23]. The remaining features can be assigned as indicated in Figure 6B, based on comparison with other known neutral flavin semiquinone radicals [23]. The H8α protons constitute the methyl group attached at C(8) and these give rise to an axial hyperfine coupling with A_⊥_ = 8.5 MHz and A_||_ = 9.7 MHz. The H6 hyperfine coupling, normally assigned as being indicative of the isotropic hyperfine coupling [23], is 5.7 MHz.

## Discussion

BluB proteins from *Rhodobacter capsulatus* and *Bacillus megaterium* have been identified.

BluB proteins from both *R. capsulatus* and *B. megaterium* were identified by comparison to the *S. meliloti* BluB. Surprisingly the results of this exercise highlighted two possible candidates for a BluB orthologue in *B. megaterium*. Both genes were expressed *in trans* in a strain of *S. enterica* that required cobinamide and DMB for growth on ethanolamine. When provided with cobinamide, expression of BluB_(Bm)_ was shown to be sufficient to rescue the growth of the strain. CbiY_(Bm)_, however, proved unable to rescue DMB-dependent growth. These results show that, BluB_(Bm)_ is able to mediate the synthesis of DMB. No activity was found associated with CbiY_(Bm)_.

Using an *in vitro* HPLC assay it was also shown that isolated recombinant BluB_(Bm)_ and BluB_(Rc)_ both synthesise DMB from a reduced flavin precursor in the presence of oxygen, further demonstrating that, like the previously identified BluB proteins from *R. rubrum* [2] and *S. meliloti* [1], [10], these proteins are single-enzyme DMB synthases. These findings support the concept that a single gene product is responsible for catalysing the complex biosynthesis of DMB from FMNH_2_.

The isolation of functional BluB proteins from *R. capsulatus* and *B. megaterium* demonstrate that this enzyme is not specific to the late cobalt insertion pathway and that the single-enzyme conversion of a FMN to DMB is apparently common to organisms of both B_12_ synthetic pathways. As the processes require molecular O_2_, the anaerobic pathways employing BluB must operate in organisms that are at least microaerophilic.

### The Biosynthesis of DMB Proceeds via a 4a-peroxyflavin Intermediate

The formation of DMB is a chemically complex process involving the breakage of three bonds as well as the formation of a fourth, all of which are catalysed by a single ‘flavin destructase’ enzyme. The complexity of the process has led to speculation as to the possible mechanism of the BluB catalysed DMB biosynthesis [1], [24], [25]. All the mechanisms proposed so far share a common first stage intermediate, a peroxyflavin, thought to be formed by the interaction of molecular oxygen with the reduced flavin. The mechanism of 4a-peroxyflavin formation is strongly supported by crystallographic data demonstrating the presence of an apparent ‘peroxyanion hole’ formed by the O2′ hydroxyl group of the ribityl tail of the flavin and the backbone amide of an active site residue in BluB from *S. meliloti.* These H-bonds could serve to not only position the molecular oxygen for attack at the C4a position of the flavin but also to stabilize the peroxyflavin intermediate [1]. Using stopped-flow spectroscopy we have, for the first time, demonstrated experimentally that BluB_(Rc)_ catlaysis proceeds via a 4a-peroxyflavin intermediate. When reduced flavin and BluB_(Rc)_ are combined with molecular oxygen a characteristic, relatively short-lived peak, is observed at 380 nm.

The 4a-peroxyflavin is generated rapidly and is followed by a much slower decay phase (in the order of seconds). Peroxyflavin was not observed in reactions undertaken in the absence of oxygen, demonstrating that the formation of peroxyflavin requires molecular oxygen. The lifetime of the 4a-peroxyflavin is relatively brief in comparison to the overall rate of DMB formation, suggesting that the formation of a peroxyflavin intermediate is not the rate limiting step of in the biosynthesis of DMB. A control experiment indicated that there is no evidence for the formation of a peroxyflavin in the absence of the enzyme (Figure S1).

The formation of a peroxyflavin intermediate was not observed under similar conditions in stopped flow experiments with BluB from *B. megaterium*. It is possible that either the formation of the 4a-peroxyflavin intermediate in the case of *B. megaterium* DMB synthesis occurs over a much longer time scale than is observed with the *R. capsulatus* homolog (and thus may not accumulate if the rate of reoxidation of the flavin is relatively fast) or conversely that the formation and degradation of the 4a-peroxyflavin occurs much more rapidly in *B. megaterium*, thereby prohibiting the detection of the UV-visible signal as it occurs within the dead time of the stopped-flow instrument. Considering that the overall rate of formation of DMB for BluB_(Bm)_ was found to be slower than that observed for BluB_(Rc)_, the former explanation is the more likely.

### CbiY from *Bacillus Megaterium* is not a BluB Protein

Interestingly, when searching for the BluB-encoding gene in the genome of *B. megaterium*, in addition to BluB_(Bm),_ another gene with high sequence similarity to known BluB proteins was also identified (Table S1). This gene *cbiY* is located on the *cobI* operon and was therefore implicated in cobalamin biosynthesis in *B. megaterium* [11].

The experimental results reported herein have demonstrated that CbiY is not a DMB synthase. It is unable to rescue the DMB-dependent growth of a transformed *S. enterica* strain, and it lacks the ability to utilise FMN to form DMB *in vitro*. In addition, unlike any BluB proteins characterised to date, it was found to bind a flavin cofactor. The flavin cofactor could be removed from the holoenzyme by denaturing the protein by heat treatment, indicating that it is non-covalently bound, and was identified as FMN (Figure S4). CbiY was shown to stabilize a neutral, blue semiquinone species when reduced anaerobically with sodium dithionite. The H8α hyperfine coupling determined from the W-band ENDOR experiment is large, A_iso_ = 8.9 MHz, compared to typical H8α hyperfine couplings in protein-bound neutral flavin semiquinones of 7–7.4 MHz. A recent study [26] has attributed this phenomenon to solvent exposure of the dimethylbenzene portion of the flavin. Such a flavin environment is typical of flavodoxins that act as ‘promiscuous’ single electron transfer proteins, stabilising the flavin semiquinone [27].

CbiY is first the flavoprotein to be identified in the *cobI* operon of *B. megaterium*. Other examples of flavoproteins involved in the biosynthesis of B_12_ have been previously reported in bacterial species utilising the late cobalt insertion pathway of B_12_ synthesis, for example CobZ and CobR. CobZ from *R. capsulatus* is an FAD-binding mono-oxygenase involved in the ring contraction step of cobalamin biosynthesis. CobZ, however, unlike CbiY, shows no stabilization of a flavin semiquinone [28]. Although CobR, a flavoprotein from *Brucella melitensis*, exhibits co(II)rrin reductase activity by a mechanism that proceeds via a single electron transfer, no stable semiquinone is observed with this enzyme [29]. The stabilization of a neutral radical species, although well documented in several flavoproteins [30], [31], [32], would be none the less novel for an enzyme implicated in B_12_ synthesis. Furthermore it is unusual to observe such substantial formation of a semiquinone species, particularly considering that the redox potentials of the ox/sq and sq/hq transitions are only ∼60 mV apart, indicating that the reduction of the flavin cofactor is possibly kinetically (rather than thermodynamically) controlled. This unusual stabilization of a radical species suggests that CbiY may be involved in a single-electron transfer pathway. However, no experimental evidence for a reductase role for CbiY has yet been obtained; it is not considered to be a flavin reductase because it does not reduce flavin in the presence of NADH (data not shown).

Thus it is unclear, as yet, what role CbiY may perform *in vivo*. The gene is located in a cobalamin biosynthetic operon, however, no direct requirement for CbiY in the biosynthesis of cobalamin has ever been demonstrated. On the basis of this evidence it is plausible that CbiY is not involved in the *de novo* synthesis of cobalamin but instead could play a role in another downstream cobalamin dependent process. This type of arrangement, although in reverse, has been observed with the _L_-threonine kinase enzyme (PduX) which initiates the synthesis of aminopropanol through the phosphorylation of threonine [33]. Aminopropanol is required for the biosynthesis of cobalamin, since it is used to create the linker to which DMB is ultimately attached. The surprise with PduX was that the encoding gene was located in the 1,2-propanediol utilisation (PDU) operon. However, the degradation of propanediol is cobalamin dependent process. Although we cannot currently propose a role for CbiY, the data obtained in this work are a crucial first step in the characterization of the mechanism of CbiY, and in turn, may be used to help elucidate the true function of this flavoprotein.

## Materials and Methods

### Chemicals and Reagents

Chemicals were purchased from Sigma-Aldrich if not mentioned otherwise. Other materials and reagents were provided by Promega UK (restriction and modification enzymes, the vector pGEM®-T easy), New England Biolabs UK (restriction and modification enzymes), Novagen (the vector pET14b), Amersham Biosciences UK (chelating Sepharose fast flow resin), Oxoid UK (tryptone, yeast extract and agar-agar), Difco (nutrient broth), Fisher/Invitrogen UK (oligomers).

### Molecular Biology Techniques

Strains and plasmids used in this work are given in Tables 1 and 2. *Escherichia coli* strain JM109 (Promega) was utilized for all DNA manipulations according to established laboratory methods [34]. The QIAprep® Miniprep kit (Qiagen) and the Wizard Plus SV Plasmid Miniprep kit (Promega) were used to isolate plasmid DNA. DNA fragments were extracted from 0.7–1% (w/v) agarose gels using a QIAquick gel extraction kit (Qiagen). PCR products were purified using a QIAquick PCR purification kit (Qiagen) or the Wizard SV Gel and PCR Clean-Up System kit (Promega). Cloned DNA fragments were sequenced by GATC Biotech (Germany) or at the Biotechnology Center of the University of Wisconsin (Madison, USA).

**Table 1:** Strains used in this study.

Strain	Genotype	Reference or source
*Escherichia coli*		
JM109	*end*A1, *rec*A1, *gyr*A96, *thi, hsd*R17 (r_k_^–^, m_k_^+^), *rel*A1, *sup*E44, Δ(*lac-pro*AB), [F′ *tra*D36, *pro*AB, *laq*I^q^ZΔM15]	Promega
BL21star(DE3)pLysS	F^–^, *omp*T, *hsd*SB(r_B_^–^, m_ B_^–^), *gal*, *dcm*, *rne*131, (DE3), pLysS (Cm^r^)	Invitrogen
DH5α/F’	F’/*endA1 hsdR17* (r_k_^−^m_k_^+^) *glnV44 thi-1 recA1 gyrA* (Nal^R^) *relA1* Δ(*lacIZYA-argF*)U169 *deoR* (φ80dlacΔ(*lacZ*)M15)	[42]
*Salmonella enterica*		
JE2119	*S. enterica metE205 ara-9 cobC1175*::Tn10d[*tet* ^+^]	J. Escalante-Semerena lab collection
JE7087	*S. enterica metE2701*::*cat* ^+^ *ara-9*	J. Escalante-Semerena lab collection
JE9384	*S. enterica metE2701*::*cat* ^+^ *ara-9*/pBluB9_(Rr)_ (*R. rubrum BluB* ^+^ *bla* ^+^)	This work
JE9385	*S. enterica metE2701*::*cat* ^+^ *ara-9*/pBAD24 (*bla* ^+^)	This work
JE12053	*S. enterica metE2701*::*cat* ^+^ *ara-9*/pBluB25_(Bm)_ (*B. megaterium BluB* ^+^ *bla* ^+^)	This work
JE12130	*S. enterica metE2701*::*cat* ^+^ *ara-9*/pCbiY6_(Bm)_ (*B. megaterium cbiY* ^+^ *bla* ^+^)	This work
JE10515	*S. enterica metE205 ara-9 cobC1175*::Tn10d[*tet* ^+^]/pBAD24 (*bla* ^+^)	This work
JE10516	*S. enterica metE205 ara-9 cobC1175*::Tn10d[*tet* ^+^]/pBluB9_(Rr)_ (*R. rubrum BluB* ^+^ *bla* ^+^)	This work
JE12054	*S. enterica metE205 ara-9 cobC1175*::Tn10d[*tet* ^+^]/pBluB25_(Bm)_ (*B. megaterium BluB* ^+^ *bla* ^+^)	This work
JE12131	*S. enterica metE205 ara-9 cobC1175*::Tn10d[*tet* ^+^]/pCbiY6_(Bm)_ (*B. megaterium cbiY* ^+^ *bla* ^+^)	This work

**Table 2:** Plasmids used in this study.

Plasmid	Description	Reference or source
pET14b	N-terminal his_(6)_-tag sequence followed by thrombin site, recombinant gene expressionunder control of T7 RNA dependant promoter, amp^r^	Novagen
pGEM®-T Easy	PCR product cloning vector	Promega
pBAD24	recombinant gene expression under control of the P_BAD_-promoter, amp^r^	[17]
pET14b-BluB(Bm)	*BluB*_(*Bm*)_ BglII/XhoI cloned with pET14b BamHI/XhoI, amp^r^	This work
pGEM®-T Easy-BluB(Rc)	*BluB*_(*Rc*)_ cloned with pGEM®-T Easy, amp^r^	This work
pET14b-BluB(Rc)	*BluB*_(*Rc*)_ NdeI/BamHI cloned with pET14b NdeI/BamHI, amp^r^	This work
pET14b-cbiY(Bm)	*cbiY*_(*Bm*)_ NdeI/BamHI cloned with pET14b NdeI/BamHI, amp^r^	This work
pBluB9_(Rr)_	*BluB_(Rr)_* EcoRI/XbaI cloned with pBAD24 EcoRI/XbaI, amp^r^	This work
pBluB25_(Bm)_	*BluB*_(*Bm*)_ EcoRI/HindIII cloned with pBAD24 EcoRI/HindIII, amp^r^	This work
pCbiY6_(Bm)_	*cbiY*_(*Bm*)_ EcoRI/XbaI cloned with pBAD24 EcoRI/XbaI, amp^r^	This work

### Cloning of *bluB* from *Rhodobacter capsulatus*, of *bluB* and *cbiY* from *Bacillus megaterium* and of *bluB* from *Rhodospirillum rubrum*

The *R. capsulatus bluB* was PCR amplified from genomic DNA from *R. capsulatus* strain SB1003 with primers containing restriction sites for *NdeI* and *BamHI* (given in *italics*). The sequence of the forward primer was 5′ ca*catatg*aactttgaacagacc 3′ and that of the reverse primer was 5′ cct*ggatcc*gttagcgccgctcccattcgg 3′. The amplified fragment was cloned initially into pGEM®-T easy (Promega). For pET14b-BluB(Rc), the gene was then subcloned from this new plasmid into pET14b (Novagen) cut with *Nde*I and *Bam*HI. *BluB* from *B. megaterium* was amplified by PCR with genomic DNA from *B. megaterium* strain DSM319 as a template. The primers used contained the restriction sites for *Bgl*II and *Xho*I (given in *italics*) and the sequences were 5′ cat*ctcgag*aattcgtttacaaacgacg 3′ and 5′ cat*agatct*ctattctttttcattttccc 3′. The resulting PCR product was cloned into the *Bam*HI/*Bgl*II digested pET14b (Novagen) to yield pET14b-BluB(Bm). For pBluB25_(Bm)_, the coding sequence of *bluB* from *B. megaterium* was PCR amplified from plasmid pET14b-BluB(Bm) as a template. The primers contained the restriction sites for *Eco*RI and *Hin*dIII (given in *italics*) and had the sequences 5′ atg*gaatcc*atgaactcgtttacaaacgacg 3′ and 5′ agt*aagctt*ctattctttttcattttcccataca 3′. The resulting product encoding BluB with a silent T6C mutation eliminating the natural *Eco*RI site in the gene, was cloned into the *Eco*RI and *Hin*dIII sites of plasmid pBAD24 [17] to yield plasmid pBluB25_(Bm)_. The *cbiY* gene from *B. megaterium* was amplified by PCR using genomic DNA from *B. megaterium* strain DSM319 as a template. The restriction sites for *Nde*I and *Bam*HI were inserted into the primers (given in *italics*). The primer sequences were 5′ gc*catatg*acgattatctcacagc 3′ and 5′ gc*ggatcc*cagtttacagccatgttg 3′. For pET14b-cbiY(Bm), the PCR fragment was finally cloned into the *Nde*I and *Bam*HI sites of pET14b (Novagen). For pCbiY6_(Bm)_, the coding sequence of *cbiY* from *B. megaterium* was amplified by PCR using genomic DNA from *B. megaterium* strain DSM319 as a template. The primers contained the restriction sites for *Eco*RI and *Xba*I and had the sequences 5′ atg*gaattc*atgacgattatctcacagctaaaag 3′ and 5′ atg*tctaga*ttacagccatgttgttttttctg 3′. The resulting product was cloned into the *Eco*RI and *Xba*I sites of plasmid pBAD24 to yield plasmid pCbiY6_(Bm)_. The *bluB* gene from *R. rubrum* was amplified by PCR using genomic DNA from *R. rubrum* UR2 as a template. The sequence of the forward primer, containing an *Eco*RI restriction site, was 5′ actgaa*gaattc*atgcgcaccggtcccctttt 3′, and that of the reverse primer, containing an *Xba*I site, 5′ actgaa*tctaga*ttaacgccgcaccaccggat 3′. The *Eco*RI/*Xba*I digested PCR product was cloned into *Eco*RI/*Xba*I digested pBAD24 to yield pBluB9_(Rr)_.

### Overproduction of Recombinant Proteins and Protein Purification Techniques

For the overproduction of recombinant proteins, *E. coli* strain BL21star(DE3)pLysS was transformed with the appropriate pET14b derivative. The recombinant strain was grown under aerobic conditions at 37°C in Luria Bertani (LB) medium supplemented with 100 mg l^−1^ ampicillin and 34 mg l^−1^ chloramphenicol. When the cells reached an optical density (OD) at 578 nm of ∼0.6, the recombinant protein overproduction was induced by the addition of isopropyl-1-thio-β-d-galactopyranoside (IPTG) to a final concentration of 400 µM and the cells were grown overnight at 19°C. They were harvested by centrifugation (3,500×g for 15 min and 4°C, Beckmann Coulter, JLA-9.1000). The cell pellets were resuspended in binding buffer (20 mM Tris pH 8.0 containing 500 mM NaCl and 10 mM imidazole). Cells were lysed by sonication (Sonics Vibracell Ultrasonic processor). After removal of the cell debris by centrifugation at 35,000×g for 20 min and 4°C, the resulting supernatant was loaded onto a metal affinity chromatography column charged with divalent Ni^2+^ and pre-equilibrated with binding buffer. After washing the column with 20 mM Tris (pH 8.0) buffer containing 500 mM NaCl and 10 mM, 50 mM, and finally 100 mM imidazole, the recombinant his_6_-tagged protein was eluted with 400 mM of imidazole. Elution fractions containing high amounts of overproduced protein were desalted by gel filtration using a Sephadex G25 PD10 column (GE Healthcare) equilibrated in 20 mM Tris (pH 8.0) containing 100 mM NaCl. Proteins were purified in the presence of oxygen at room temperature and then kept on ice. Fractions were analyzed by SDS-polyacrylamide gel electrophoresis (PAGE) according to the method described by [35]. The gel was stained with coomassie brilliant blue [34]. Size standard was Dalton VII (Sigma-Aldrich) with marker proteins of 66, 45, 36, 29, 24, 20.1 and 14.2 kDa.

### Fast Protein Liquid Chromatography (FPLC)

The desalted protein fractions were loaded onto a pre-packed high flow, high resolution Superdex 200 column (10/300 GL) equilibrated in Buffer A (20 mM Tris-HCl (pH 8.0) containing 100 mM NaCl). The proteins were eluted at 0.5 ml min^−1^ into 1 ml fractions with buffer A on an Akta® FPLC chromatography system at 19°C. Fractions were analyzed by SDS-PAGE (Läemmli 1970) and the purest fractions were pooled.

### BluB *in vitro* Activity Assay

The formation of dimethylbenzimidazole (DMB) was monitored in 1 ml reactions containing 100 µM flavin adenine mononucleotide (FMN) and 40 mM nicotinamide adenine dinucleotide (reduced form) (NADH) in 20 mM Tris-HCl (pH 8.0) containing 100 mM NaCl and 1 mM dithiothreitol (DTT) at room temperature in the presence of oxygen in the dark for 18 h. The amount of the purified enzymes in the reaction mixtures varied between 10 µg and 4.8 mg. Control reactions were performed in the absence of either FMN, NADH, or enzyme. Reactions were stopped by boiling the samples for 5 min followed by a centrifugation step at 14,000 rpm at 4°C for 10 min. The products of the reaction were separated and identified by high performance liquid chromatography on an ACE 5 AQ column (4.6×250 mm; Advanced Chromatography Technologies) run on an Agilent 1100 series HPLC equipped with a diode array detector. Intermediates were eluted with a flow rate of 1 ml min^−1^ and a gradient of methanol in 5 mM ammonium acetate pH 6.5 as described previously [2]. DMB and FMN were identified by comparison of their absorbance spectra and retention time compared with authentic DMB and FMN standards.

### Formation of the BluB Peroxyflavin Intermediate

After aerobic overproduction and purification of the recombinant BluB enzymes, the elution fractions where transferred into an anaerobic glove box (Bell Technology), maintained at less than 2 ppm oxygen. The protein was desalted and liberated from oxygen by gel filtration using a Sephadex G25 PD10 column (GE Healthcare) equilibrated in oxygen free 20 mM Tris-HCl (pH 8.0) containing 100 mM NaCl. The protein (42–500 µM) was pre-incubated with 171 µM reduced FMN (produced by the reduction of FMN with sodium dithionite (DT)) and rapidly mixed with non-degassed (air saturated) buffer in an Applied Photophysics SX20 stopped-flow spectrometer at 20°C. The reaction was monitored by photodiode array detector. Formation of the peroxyflavin derivative, with an absorption maximum at 380 nm, was complete with in 20 ms of mixing.

### Identification of Flavin Cofactor Bound to CbiY

The bound flavin cofactor was extracted from CbiY_(Bm)_ by incubation of the protein at 100°C for 10 minutes. The precipitate was removed by centrifugation leaving a yellow supernatant, 10 µl of which was injected on to an Ace 5 AQ column (4.6×210 mm, 5 µm, Advanced Chromatography Technologies) run on an Agilent 1100 series HPLC equipped with online diode array and fluorescence detectors linked to a Bruker micrOTOF-Q II mass spectrometer at a flow rate of 0.2 ml min^−1^. Flavin cofactors were separated isocratically using 25% (v/v) methanol in 20 mM ammonium bicarbonate (pH 7.5) as the eluent. The absorbance at 455, fluorescence emission at 525 nm (excitation wavelength 445 nm) and the mass spectrum was monitores. Identification of species was based on the comparison of the retention time and mass spectrum with that of commercial standards.

### Determination of Flavin Redox Potentials

Potentiometric redox titrations were performed to determine the midpoint reduction potentials for the flavin cofactor housed within CbiY _(Bm)_. Spectroelectrochemical potentiometric titrations were performed in a anaerobic glove box (Belle Technology) under a nitrogen atmosphere as initially described by Dutton and as detailed in our previous studies of other flavoproteins [36], [37], [38]. Absorption *versus* potential data were fitted at various wavelength to establish the reduction potentials for both the 2-electron reduction couple of the CbiY flavin (oxidized/hydroquinone, *E*_12_), and for the individual oxidized/semiquinone (ox/sq) (*E*_1_) and semiquinone/hydroquinone (sq/hq) (*E*_2_) couples. Data were fitted at both 443 nm (at the oxidized flavin maximum) and at 465 nm (at the major oxidized flavin shoulder), and at 487 nm (isosbestic point for the flavin ox/sq transition) and at 430 nm (isosbestic point for the flavin sq/hq transition). Data were fitted using 1- and 2-electron Nernst equations, as described previously [39].

### EPR and ENDOR Spectroscopy

EPR and ENDOR spectra were obtained using either a Bruker ELEXSYS E500/580 EPR spectrometer operating at X-band or a Bruker ELEXSYS E600/680 EPR spectrometer operating at W-band. Temperature control was effected using Oxford Instruments ESR900, CF935 and CF1200 cryostats interfaced with an ITC503 temperature controller. Experimental conditions were as given in the figure caption.

### Complementation Assays

*S. enterica* strains were grown aerobically overnight in nutrient broth supplemented with 50 µg µl^−1^ ampicillin. For derivatives of *S. enterica* strain JE7087, overnight cultures were subcultured (1∶50 vol:vol inoculum) into no-carbon E (NCE) medium [40] supplemented with 90 mM ethanolamine, 1 mM MgSO_4_, 1× trace minerals [41] (53 µM nitrilotriacetic acid, 12.5 µM MnSO_4_ • 4H_2_O, 171 µM NaCl, 4 µM FeSO_4_ • 7H_2_O, 4 µM CoSO_4_, 7 µM CaCl_2_ • 2H_2_O, 3.5 µM ZnSO_4_, 0.4 µM CuSO_4_ • 5H_2_O, 2 µM H_3_BO_3_, 12 µM Na_2_MoO_4_ • 2H_2_O, 11 µM Na_2_SeO_4_ • 10H_2_O, 2 µM NiSO_4_ • 6H_2_O), 150 nM dicyanocobinamide, and 50 µg µl^−1^ ampicillin. Aerobic growth of JE7087 derivatives took place in 200 µl volumes in 96-well microtiter dishes (Falcon). Optical density at 650 nm was measured for 36 h at 37°C. Growth curves were obtained using an ELx808 Ultra Microplate reader (Bio-Tek Instruments). For derivatives of *S. enterica* strain JE2119, overnight cultures were rinsed twice with sterile saline and subcultured (1∶50 vol:vol inoculum) into NCE medium containing 11 mM glucose, 1 mM MgSO_4_, 15 nM dicyanocobinamide, 1× trace minerals, and 50 µg µl^−1^ ampicillin. Aerobic growth of JE2119 derivatives took place in 5 ml volumes in 125 ml sidearm flasks which were agitated at 280 rpm at 37°C. Growth was monitored with a Summerson colorimeter (Klett). Each growth curve was performed in triplicate.

## Supporting Information

Figure S1 **Control stopped flow experiment.** FMNH_2_ (291 µM) combined in the stopped flow with oxygenated buffer A. Spectra were collected every 2.5 s. An increase in absorbance at 375 nm and 460 nm (red) indicate a reoxidation of free FMN. This was followed by a loss of absorbance (black) over time probably due to photobleaching of the flavin.
(DOC)

Figure S2 **Subtraction spectrum.** Dashed line shows the initial spectrum of BluB and FMNH_2_ combined with oxygenated buffer (291 µM FMNH_2_ was incubated with 684 µM (RC)BluB and prior to stopped flow mixing with oxygenated buffer A. Spectrum recorded 2.5 s after mixing), dotted line shows the initial spectrum of the control sample (291 µM FMNH_2_ combined in the stopped flow with oxygenated buffer A. Spectrum recorded 2.5 s after mixing) and the solid line shows the resulting spectrum if the control spectrum is subtracted from the sample spectrum.
(DOCX)

Figure S3 **Rates of peroxyflavin formation and degradation; variable BluB concentration.** The figure shows the apparent rate of the formation of c4a-peroxyflavin in the presence of a variable concentration of (RC)BluB (black circles). The concentration of FMNH_2_ remained constant at 171 µM and the absorbance was measured at 380 nm. The degradation of the peroxyflavin species appears to be biphasic, the two apparent rates are also shown here (white circles and black triangles). Temperature was maintained at 25°C throughout.
(DOC)

Figure S4 **Analysis of the flavin cofactor bound to CbiY.** HPLC chromatogram and ESI +ve mass spectrum of the flavin cofactor isolated from CbiY. Retention time and MS were consistent with the bound cofactor being FMN (m/z 457 [M+H]^+^).
(DOCX)

Table S1 **Analysis of sequence alignments.** The table shows the consensus sequence given in [%] and the identity given in [%] of the BluB proteins from *R. capsulatus*, *R. rubrum*, *S. meliloti*, and *B. megaterium* (**A**) and consensus sequence given in [%] and identity given in [%] of CbiY from *B. megaterium* compared with BluB proteins of *R. capsulatus*, *R. rubrum*, *S. meliloti*, and *B. megaterium* (**B**).
(DOC)
