# Supplementary material for: Bacillus megaterium Has Both a Functional BluB Protein Required for DMB Synthesis and a Related Flavoprotein That Forms a Stable Radical Species
Source: PLoS One. 2013 Feb 14;8(2):e55708. doi: 10.1371/journal.pone.0055708 (PMC3573010; doi:10.1371/journal.pone.0055708)
Supplement: Figure S1 — Control stopped flow experiment. FMNH2 (291 µM) combined in the stopped flow with oxygenated buffer A. Spectra were collected every 2.5 s. An increase in absorbance at 375 nm and 460 nm (red) indicate a reoxidation of free FMN. This was followed by a loss of absorbance (black) over time probably due to photobleaching of the flavin. (DOC) [file pone.0055708.s001.doc]

**Figure S1 Control stopped flow experiment**


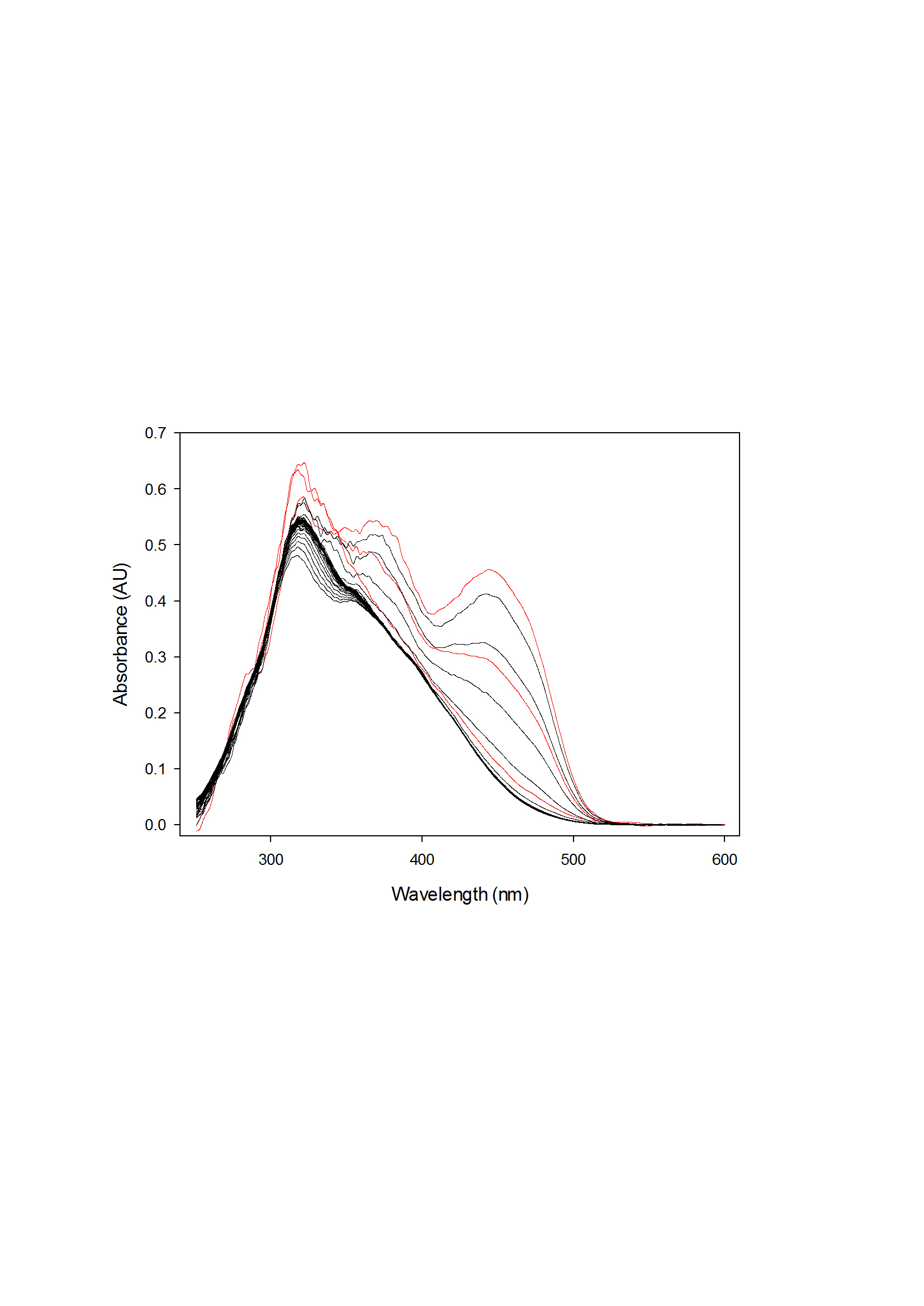


291 µM FMNH2 combined in the stopped flow with oxygenated buffer A. Spectra were collected every 2.5 s. An increase in absorbance at 375 nm and 460 nm (red) indicate a reoxidation of free FMN. This was followed by a loss of absorbance (black) over time probably due to photobleaching of the flavin.
