# Supplementary material for: Bacillus megaterium Has Both a Functional BluB Protein Required for DMB Synthesis and a Related Flavoprotein That Forms a Stable Radical Species
Source: PLoS One. 2013 Feb 14;8(2):e55708. doi: 10.1371/journal.pone.0055708 (PMC3573010; doi:10.1371/journal.pone.0055708)
Supplement: Figure S2 — Subtraction spectrum. Dashed line shows the initial spectrum of BluB and FMNH2 combined with oxygenated buffer (291 µM FMNH2 was incubated with 684 µM (RC)BluB and prior to stopped flow mixing with oxygenated buffer A. Spectrum recorded 2.5 s after mixing), dotted line shows the initial spectrum of the control sample (291 µM FMNH2 combined in the stopped flow with oxygenated buffer A. Spectrum recorded 2.5 s after mixing) and the solid line shows the resulting spectrum if the control spectrum is subtracted from the sample spectrum. (DOCX) [file pone.0055708.s002.docx]

Figure S2


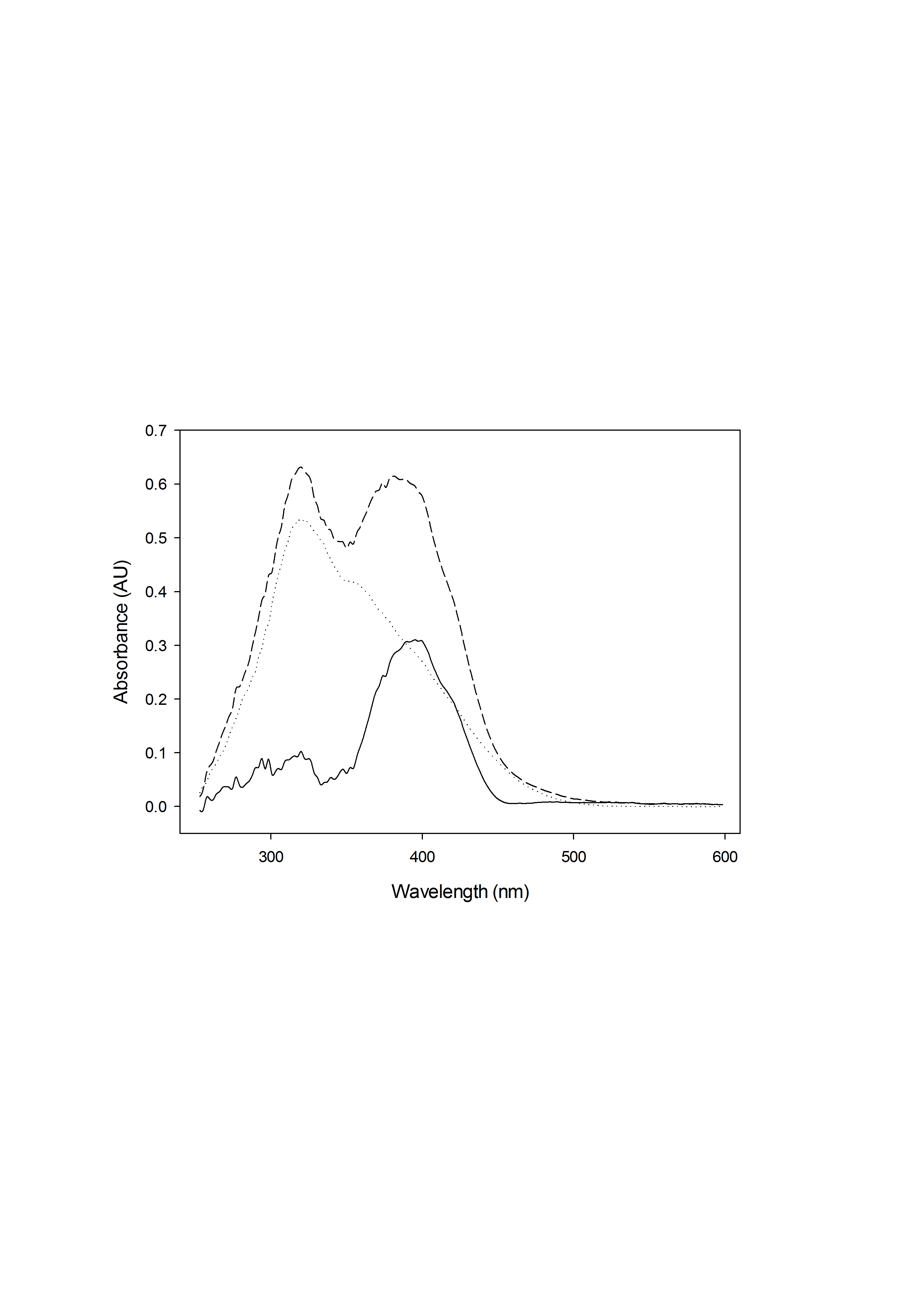


Dashed line shows the initial spectrum of BluB and FMNH_2_ combined with oxygenated buffer (291 µM FMNH_2_ was incubated with 684 µM (RC)BluB and prior to stopped flow mixing with oxygenated buffer A. Spectrum recorded 2.5 s after mixing), dotted line shows the initial spectrum of the control sample (291 µM FMNH_2_ combined in the stopped flow with oxygenated buffer A. Spectrum recorded 2.5 s after mixing) and the solid line shows the resulting spectrum if the control spectrum is subtracted from the sample spectrum.
