# Supplementary material for: Bacillus megaterium Has Both a Functional BluB Protein Required for DMB Synthesis and a Related Flavoprotein That Forms a Stable Radical Species
Source: PLoS One. 2013 Feb 14;8(2):e55708. doi: 10.1371/journal.pone.0055708 (PMC3573010; doi:10.1371/journal.pone.0055708)
Supplement: Figure S3 — Rates of peroxyflavin formation and degradation; variable BluB concentration. The figure shows the apparent rate of the formation of c4a-peroxyflavin in the presence of a variable concentration of (RC)BluB (black circles). The concentration of FMNH2 remained constant at 171 µM and the absorbance was measured at 380 nm. The degradation of the peroxyflavin species appears to be biphasic, the two apparent rates are also shown here (white circles and black triangles). Temperature was maintained at 25°C throughout. (DOC) [file pone.0055708.s003.doc]

**Figure S3 Rates of peroxyflavin formation and degradation; variable BluB concentration**

**
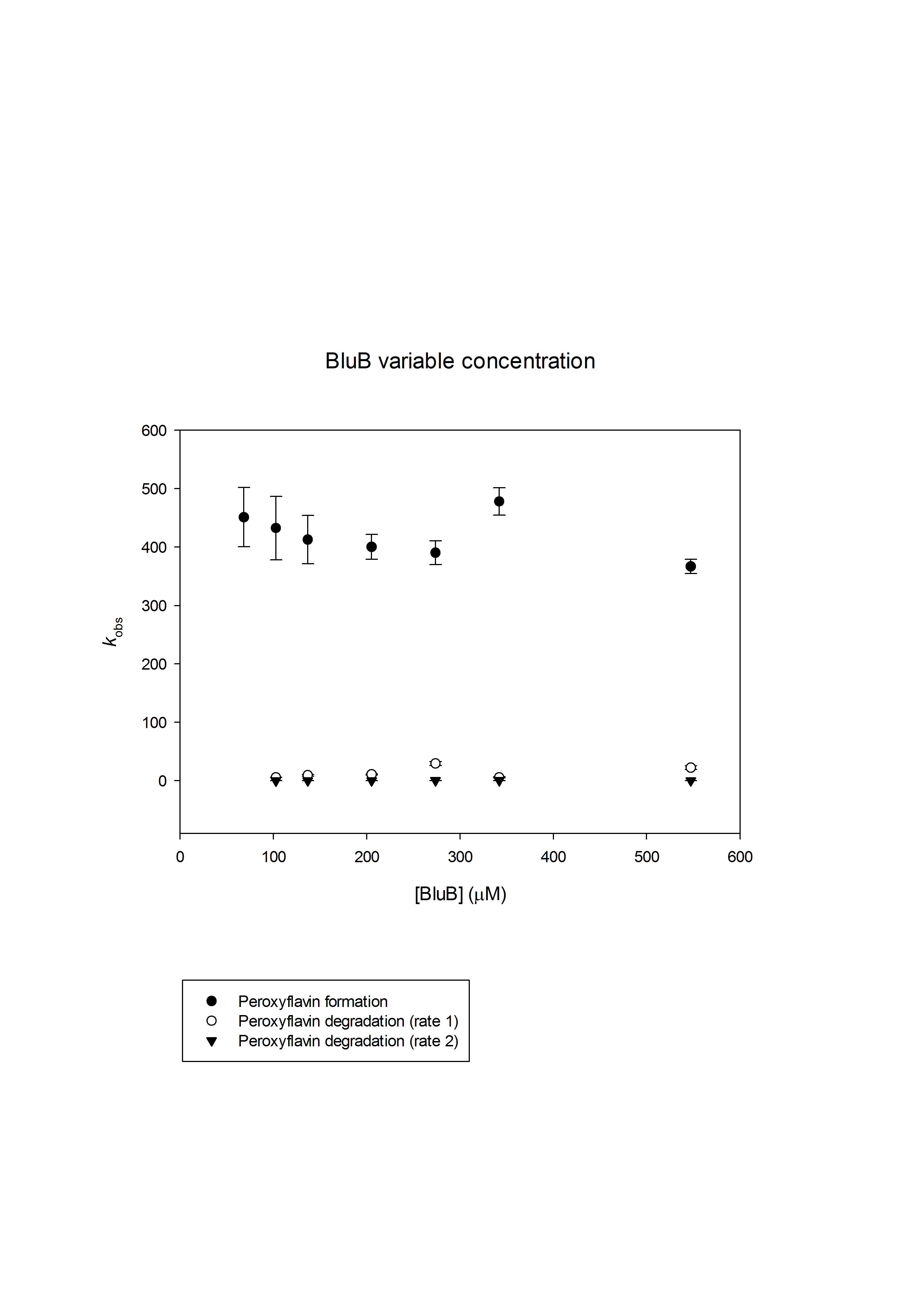
**

The figure shows the apparent rate of the formation of c4a-peroxyflavin in the presence of a variable concentration of (RC)BluB (black circles). The concentration of FMNH_2_ remained constant at 171 µM and the absorbance was measured at 380 nm. The degradation of the peroxyflavin species appears to be biphasic, the two apparent rates are also shown here (white circles and black triangles). Temperature was maintained at 25 °C throughout.
