# Supplementary material for: Bacillus megaterium Has Both a Functional BluB Protein Required for DMB Synthesis and a Related Flavoprotein That Forms a Stable Radical Species
Source: PLoS One. 2013 Feb 14;8(2):e55708. doi: 10.1371/journal.pone.0055708 (PMC3573010; doi:10.1371/journal.pone.0055708)
Supplement: Figure S4 — Analysis of the flavin cofactor bound to CbiY. HPLC chromatogram and ESI +ve mass spectrum of the flavin cofactor isolated from CbiY. Retention time and MS were consistent with the bound cofactor being FMN (m/z 457 [M+H]+). (DOCX) [file pone.0055708.s004.docx]

**Figure S4 Analysis of the flavin cofactor bound to CbiY.**

HPLC chromatogram and ESI +ve mass spectrum of the flavin cofactor isolated from CbiY. Retention time and MS were consistent with the bound cofactor being FMN (m/z 457 [M+H]^+^).
