# Supplementary material for: Bacillus megaterium Has Both a Functional BluB Protein Required for DMB Synthesis and a Related Flavoprotein That Forms a Stable Radical Species
Source: PLoS One. 2013 Feb 14;8(2):e55708. doi: 10.1371/journal.pone.0055708 (PMC3573010; doi:10.1371/journal.pone.0055708)
Supplement: Table S1 — Analysis of sequence alignments. The table shows the consensus sequence given in [%] and the identity given in [%] of the BluB proteins from R. capsulatus, R. rubrum, S. meliloti, and B. megaterium (A) and consensus sequence given in [%] and identity given in [%] of CbiY from B. megaterium compared with BluB proteins of R. capsulatus, R. rubrum, S. meliloti, and B. megaterium (B). (DOC) [file pone.0055708.s005.doc]

**Table S1.** Analysis of sequence alignments

| **(A)** |  |  |
| --- | --- | --- |
| **Comparison of BluB proteins**  **from** | **Consensus [%]** | **Identity [%]** |
| *R. rubrum + R. capsulatus* | 55.5 | 46.9 |
| *B. megaterium + R. capsulatus* | 40.6 | 29 |
| *B. megaterium + R. rubrum* | 43.2 | 30.9 |
| *B. megaterium + S. meliloti* | 58.5 | 40.2 |
| *R. capsulatus + S. meliloti* | 40.5 | 30 |
| *R. rubrum + S. meliloti* | 47.1 | 37 |
| **(B)** |  |  |
| **BluB from** | **Consensus [%]** | **Identity [%]** |
| *S. meliloti* | 29.9 | 18.6 |
| *R. rubrum* | 34.1 | 18.9 |
| *R. capsulatus* | 25.7 | 14.1 |
| *B. megaterium* | 33.6 | 20.2 |

The table shows the consensus sequence given in [%] and the identity given in [%] of the BluB proteins from *R. capsulatus*, *R. rubrum*, *S. meliloti*, and *B. megaterium* (**A**) and consensus sequence given in [%] and identity given in [%] of CbiY from *B. megaterium* compared with BluB proteins of *R. capsulatus*, *R. rubrum*, *S. meliloti*, and *B. megaterium* (**B**).
